# Chances and limitations of nanosized titanium dioxide practical application in view of its physicochemical properties

**DOI:** 10.1186/s11671-015-0753-2

**Published:** 2015-02-11

**Authors:** Janusz Bogdan, Agnieszka Jackowska-Tracz, Joanna Zarzyńska, Joanna Pławińska-Czarnak

**Affiliations:** Department of Food Hygiene and Public Health Protection, Faculty of Veterinary Medicine, Warsaw University of Life Sciences - SGGW, Nowoursynowska 159, 02-776 Warsaw, Poland

**Keywords:** Nanotechnology, Photocatalysis, Titanium dioxide, Reactive oxygen species, Self-disinfecting and self-cleaning surfaces, Bacteria

## Abstract

Nanotechnology is a field of science that is nowadays developing in a dynamic way. It seems to offer almost endless opportunities of contribution to many areas of economy and human activity, in general. Thanks to nanotechnology, the so-called nanomaterials can be designed. They present structurally altered materials, with their physical, chemical and biological properties entirely differing from properties of the same materials manufactured in microtechnology. Nanotechnology creates a unique opportunity to modify the matter at the level of atoms and particles. Therefore, it has become possible to obtain items displaying new, useful properties, i.e. self-disinfecting and self-cleaning surfaces. Those surfaces are usually covered by a thin layer of a photocatalyst. The role of the photocatalyst is most of the time performed by the nanosized titanium dioxide (nano-TiO_2_). Excitation of nano-TiO_2_ by ultraviolet radiation initiates advanced oxidation processes and reactions leading to the creation of oxygen vacancies that bind water particles. As a result, photocatalytic surfaces are given new properties. Those properties can then be applied in a variety of disciplines, such as medicine, food hygiene, environmental protection or building industry. Practically, the applications include inactivation of microorganisms, degradation of toxins, removing pollutants from buildings and manufacturing of fog-free windows or mirrors.

## Review

### Introduction

The past decade redounded the discovery that many materials used in a number of industries - e.g. titanium white (TiO_2_) and zinc white (ZnO) applied in the manufacturing of paints and varnishes, refractory magnesia (MgO) added to cement, or silica (SiO_2_) applied in the fabrication of glass products - after they have been powdered to nanoparticles (NPs) (1 < *φ* ≤ 100 nm), significantly alter their properties, i.e. they exhibit increased hardness, tensile strength, plasticity [1], higher resistance to chemical agents [2], greater hydrophilicity [3] or (photo)catalytic properties [4]. Materials powdered to NPs, called nanomaterials (NMs), have found many applications, e.g. in the production of cosmetics [5], including sunscreens [6], fabrication of ceramics [7] and ingredients of photocatalytic coatings applied on various work surfaces [4]. On surfaces with a thin layer of photocatalyst, i.e. nanosized titanium dioxide (nano-TiO_2_), the inactivation of microorganisms [8,9] and the mineralization of organic matter [10,11] have been observed. It is a result of advanced oxidation processes (AOPs), initiated by ultraviolet (UV) radiation [12]. Photocatalytic surfaces, also referred to as self-disinfecting and self-cleaning surfaces, are applied in many fields of industry, such as the building sector (i.e. in the manufacturing of concrete blocks, plasters, windows and ceramic tiles) [13] or road transport (i.e. in the production of asphalt and road signs) [14]. Due to their antibacterial [15-17], antifungal [18,19] and deodorizing [20] properties, photocatalytic films are more and more frequently applied to coat surfaces of sanitary products, laboratory tables, air filters, as well as in hospital rooms, canteens, production halls and rooms exposed to onerous odours, e.g. animal stables. NMs can also assist in keeping clean various textile materials, such as garment, bed sheets and carpets [21,22]. Increasingly frequent are the opinions that NMs are very soon going to play a crucial role in medicine [23] as well as in a number of industries, including the pharmaceutical industry [24] and food processing [25], as weapons to destroy cancer cells and fight pathogens, as carriers of drugs in organisms or as agents modifying organoleptic parameters of food. It is assumed that the introduction of NMs in food industry, animal production, or medicine will contribute towards a more efficient prevention of food poisonings and food infections and will increase the animal welfare by creation of better living conditions for animals. It will also enhance the efficiency of antibiotic-based therapies.

There are different NMs applied to create photocatalytic coatings that confer on the surfaces self-cleaning and self-disinfecting properties. Among them, there are oxides of some metals, e.g. titanium dioxide (TiO_2_), powdered to NPs [26-32]. Its undeniable attributes are low production costs, insolubility in most of the reaction environments and high photochemical stability [33]. This oxide exists in three polymorphic forms: anatase, brookite and rutile, whereby only two of them, anatase and rutile, are widely used to obtain NMs [34].

The number and variety of goods on the market that contain nanosized metal oxides, particularly nano-TiO_2_, are immense [35]. Therefore, the level of human exposure to their effects is varied. The omnipresence of nano-TiO_2_ in the human environment may evoke justified concerns as to the potential impact of its catalytic properties on human health [36-38].

### Toxicity of titanium dioxide to people and natural environment

Since the beginning of the twentieth century, there has been a greater industrial interest in TiO_2_. Then, this pigment started to be used, replacing toxic lead compounds applied in manufacturing of paints and varnishes. As the report of the Institute for Market Research Ceresana [39] indicates, the world production of TiO_2_ - which, powdered to microparticles (MPs) (0.1 < *φ* ≤ 100 μm), is an odourless amorphic powder with a pristine white colour - amounted to 6.2 million tonnes in 2013. Its application as a white pigment is quite varied. This compound is applied in the production of paints [40], plastics [41], sunscreen cosmetics [42] and foods (i.e. as pigment E-171) [43].

The issue of TiO_2_ toxicity has been a research subject in many research centres around the world for many years. It is assumed that the microsized titanium dioxide (micro-TiO_2_) is harmless to people and animals [36,44]. As the dynamic development of nanotechnology goes on, however, there has been an increased concern that this compound might be toxic in the NP form, though. There are studies proving that NPs of many other metal oxides, such as ZnO or MgO, are more harmful to people and animals than MPs of the same compounds [45]. One of the main differences between micro-TiO_2_ and nano-TiO_2_ is a much bigger active surface of nano-TiO_2_, a feature resulting in a higher absorption rate of UV and a greater photocatalytic activity. Hence, nano-TiO_2_, contrary to micro-TiO_2_, was classified in the 2B group by the International Agency for Research on Cancer (IARC) [46]. The 2B group assembles compounds that might be carcinogenic for humans (to compare: in the same group, there are also chloroform, nitrobenzene, fumonisins B1 or ochratoxins A).

Yamamoto et al. [47] suggest that the nano-TiO_2_ present in sunscreen cosmetics (with UV filter) enhances the creation of reactive oxygen species (ROS) in skin cells, a phenomenon that may result in DNA damages and mutations and consequently induce the development of cancer diseases. Dunford et al. [48] and Subrahmanyam et al. [49] share that opinion. In view of those findings, a question as to the safety of products containing nano-TiO_2_ must be thoroughly answered [50].

In line with the Regulation (WE) 1223/2009 of the European Parliament and the Council [51], the maximum allowed amount of TiO_2_ in toiletries may not exceed 25%. As Hansen [37] calculated, the consumers' exposure to nano-TiO_2_ in face moisturizer creams, body care lotions or antiperspirants amounts annually to 26, 15 and 44 μg kg^−1^ body weight, respectively. A number of cosmetics manufacturers started to respond to consumers' fears and launched products containing nano-TiO_2_ in a new, safer formula. Effectively, the surface of nano-TiO_2_ particles is coated by a thin layer of ethylene glycol, and the particles are subsequently heated up to 300°C. In this temperature, the carbonization of ethylene glycol takes place. Therefore, the surface of nano-TiO_2_ particles becomes enfolded by a thin layer with a high carbon share that nearly completely stops their photocatalytic properties [5], without simultaneously altering their other physicochemical properties. It means that nano-TiO_2_ modified in this way is safe for the human skin. For example, when added to sunscreens, it can effectively protect the skin from negative effects of sun radiation, without generating any ROS at the same time. It seems, therefore, that the real risk of health loss due to cosmetics containing nano-TiO_2_ is scarce [50].

There are still very few studies available that would handle the issue of natural environment infiltration by nano-TiO_2_, its accumulation in water and soil, and its impact on organisms living in those miscellaneous environments [52]. According to the report by the United States Environmental Protection Agency (USEPA) [53], TiO_2_ is freed to the atmosphere, surface waters and soil, i.e. from such sources as already mentioned sunscreens, sun protecting textiles, plasters, paints or food packaging. Those data are confirmed by many authors [54-58]. Gottschalk et al. [59] present the opinion that nano-TiO_2_ occurs in a higher concentration in benthic sediments than in water. The conclusions are based on the water ecosystems studies with regard to their pollution by nanosized metal oxides. Mueller and Nowack [60] were the first to determine the quantitative risk for water animals coming from the presence of nano-TiO_2_ in their environment. By comparing the predicted effect concentration (PEC) of nano-TiO_2_ in various water habitats (0.70 to 9.60 ppb) with the predicted no effect concentration (PNEC) (less than 1.00 ppb), the authors discovered that the danger of nano-TiO_2_ for water biotope animals, including pollution-sensitive water purity indicator species (such as *Anodonta anatina*, *Heloecius cordiformis* and *Limanda limanda*), is non-existent (PEC/PNEC ≤ 1) or at most barely present (1 < PEC/PNEC ≤ 10). Having conducted their own studies, Gottschalk et al. [61] reached similar conclusions with respect to soil fauna, i.e. soil purity bioindicators (such as *Lumbricus terrestris* and *Orchesella cincta*).

Effective immobilization of nano-TiO_2_ on surfaces covered by photocatalytic films remains a crucial problem. The application of nano-TiO_2_ onto surfaces by means of magnetron sputtering deposition [62], chemical vapour deposition [63] or sol-gel deposition [64] results in an inseparably base-fixed, resistant to mechanical factors, thin layer of photocatalyst. The application of those technologies allows to significantly reduce the health damage risk, resulting from the transmission of nano-TiO_2_ from photocatalytic coatings to the environment and from the contact of free NPs, i.e. with human or animal bronchial epithelial cells.

### Photocatalytic properties of titanium dioxide

The interest in nano-TiO_2_ started with the discovery of its catalytic properties induced by UV radiation, in the beginning of the 1970s of the twentieth century [65] - and it is still growing [12,66,67]. There is already a lot of literature data presenting the mechanism of the conversion of solar energy into chemical energy and on the possibilities of increasing the photocatalysis efficiency, both homogeneous (e.g. O_3_/UV) and heterogeneous (e.g. TiO_2_/UV), in case when the catalyst is located in the same and in the other thermodynamic phase, respectively, as the substrates and products of reaction [33,68,69]. The catalytic properties of semiconductors, e.g. of TiO_2_, can be explained by their electron structure. They have a valence band (VB) full with electrons and an electron-free conduction band (CB). The energy difference (Δ*E*) between those bands, defined as band gap, also referred to as energy gap, presents the amount of energy which must be delivered in order to excite an electron from VB to CB. For three polymorphic TiO_2_ forms, brookite, anatase and rutile, the width of the energy gap amounts to 2.9, 3.0 and 3.2 eV, respectively, an equivalent of the electromagnetic radiation photon energy with a wavelength of *λ* < 400 nm. In biological experiments, to excite semiconducting metal oxides (e.g. TiO_2_), the UV radiation is used, mainly in the near-ultraviolet range (UV-A, *λ* = 315 to 400 nm) [70-73], and to a lesser extent, the UV radiation with a wavelength in the indirect ultraviolet range (UV-B, *λ* = 280 to 315 nm) [70,72]. The application of far ultraviolet (UV-C, *λ* = 100 to 280 nm) as an agent initiating AOPs poses a serious danger to human health [72].

**Figure 1:**
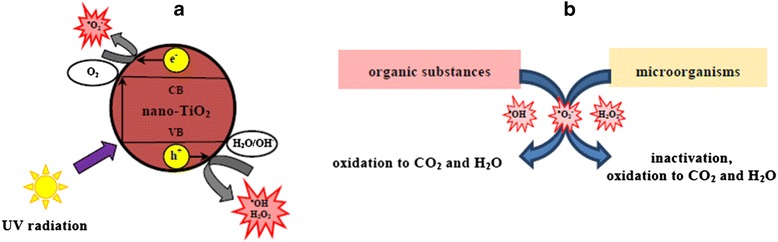
**ROS generation and its effects.** Mechanism of reactive oxygen species (ROS) generation on the surface of titanium dioxide nanoparticles **(a)** and the effects of ROS activity on organic substances and microorganisms **(b)**. On the surface of the nano-TiO_2_ particles, exposed to UV radiation, ROS (^•^O_2_^−^, ^•^OH, H_2_O_2_) are formed **(a)** that have the ability to inactivate microorganisms and to oxidize organic matter **(b)**.

As a result of the semiconductor excitation, an electron (e^−^) excites from VB to CB, leaving behind a positively charged electron hole (h^+^) hence generating a specific ‘hole-electron’ pair (h^+^ + e^−^) [33,69] (Figure 1).

This highly instable condition, called exciton, exhibits strong redox properties. The electron holes (h^+^) that condition oxidation processes, together with electrons (e^−^) that determine reduction processes, react with molecular oxygen (O_2_), water molecules (H_2_O) or hydroxyl ions (OH^−^), generating thereby ROS, such as superoxide anion radicals (^•^O_2_^−^), hydroxyl radicals (^•^OH) or hydrogen peroxide molecules (H_2_O_2_) [33,69] (Figure 1).

ROS that emerge on the photocatalytic surfaces, such as hydroxyl radicals (^•^OH), superoxide anion radicals (^•^O_2_^−^) and hydrogen peroxide (H_2_O_2_), not only inactivate but also oxidize bacteria, yeasts and moulds to CO_2_ and H_2_O [18,19,74,75], similarly as molecules of nearly all organic compounds [11,76] (Figure 1). Among the mineralized compounds, there are hydrocarbons, alcohols, aldehydes, ketones, and aromatic compounds [33,69] which are dangerous to human health, as well as dioxins and polychlorinated biphenyls (PCBs) [77,78] which are present in air and wastewater. Oxidized organic compounds are often onerous and hard to remove from air [79], water [80] and solid surfaces (e.g. windows, building exteriors and machines) [75], whereby inactivated microorganisms are frequently pathogens which are dangerous for humans and animals [81]. Due to photocatalytic properties, TiO_2_ is applied, after powdering to NPs, in AOPs and UV radiation-based methods for pathogen inactivation and organic pollutant decomposition [82].

### Superhydrophilicity and superhydrophobicity of titanium dioxide

Apart from photocatalytic properties, another unique feature of nano-TiO_2_ is strong hydrophilicity, induced by UV radiation, and called superhydrophilicity [33,67]. This mechanism provides that CB excited electrons (e^−^) reduce ions Ti^4+^ to ions Ti^3+^ (see Equation 1), while electron holes (h^+^) oxidize oxide anions (O^2−^) to molecular oxygen (O_2_) (see Equation 2). Generated oxygen molecules are removed from the nano-TiO_2_ surface. The remaining oxygen vacancies react with water molecules (H-OH). As a result, oxygen vacancies bind to the surface covered by a thin layer of nano-TiO_2_ hydroxyl ions (OH^−^) that are responsible for the surface's superhydrophilicity.

1 $$ \mathrm{T}\mathrm{i}{\mathrm{O}}_2\left({\mathrm{e}}^{-}\right)\kern0.5em +\kern0.5em \mathrm{T}{\mathrm{i}}^{4+}\to \mathrm{T}{\mathrm{i}}^{3+}\cdot $$

2 $$ 4\mathrm{T}\mathrm{i}{\mathrm{O}}_2\left({\mathrm{h}}^{+}\right)+{20}^{2-}\to {{\mathrm{O}}_2}^{\uparrow}\cdot $$

**Figure 2:**
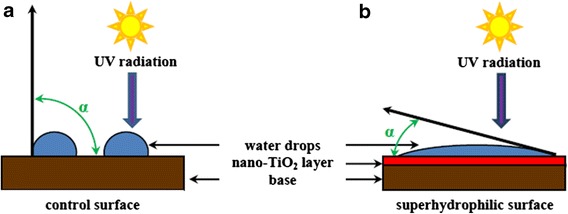
**Comparison of contact angle (** ***α*** **) on control surface (a) and surface covered by a thin layer of nano-TiO**_**2**_ **(b).** Both surfaces are exposed to UV radiation. The surface covered by a thin layer of nano-TiO_2_ and exposed to UV radiation exhibits superhydrophilic properties; the contact angle is an acute angle, 0° < *α* < 90° **(b)**.

The longer such surface is exposed to UV irradiation, the smaller the contact angle becomes, which is defined as an angle between the solid matter plane and the plane tangent to liquid drop placed on that solid matter (in the tangent point of liquid and solid matter) (Figure 2).

Thirty minutes after the exposure of the photocatalytic surface to UV radiation, the contact angle is close to zero. It means that water shows a tendency to ideally spread over the surface. It allows to obtain a thin, homogeneous, quickly evaporating, invisible and pollutant-removing film of water [83]. That is why glass surfaces covered by a thin layer of nano-TiO_2_ are applied in manufacturing of fog-free mirrors [84] or self-cleaning windows [66]. It is not the only example of the practical application of the nano-TiO_2_ strong hydrophilicity. Thanks to its superhydrophilicity, nano-TiO_2_ is also widely used in production of self-cleaning plasters [10], paints [40] and plastics [6,41].

**Figure 3:**
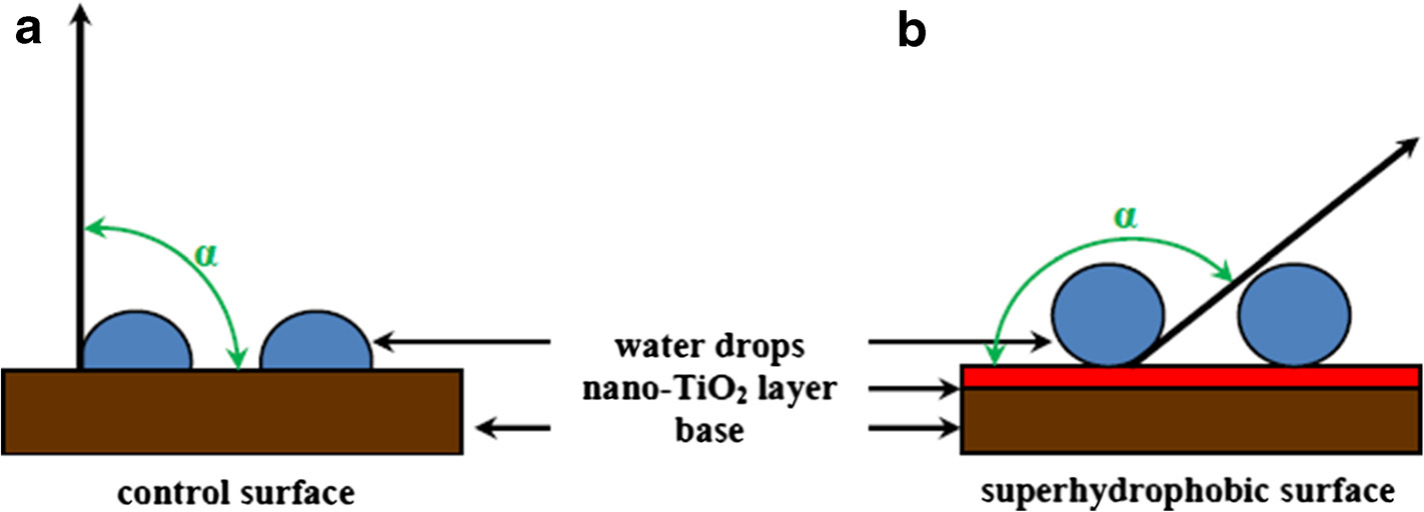
**Comparison of contact angle (** ***α*** **) on control surface (a) and surface covered by a thin layer of nano-TiO**_**2**_ **(b) in darkness.** The surface covered by a thin layer of nano-TiO_2_ exhibits in darkness superhydrophobic properties; the contact angle is an obtuse angle, 90° < *α* < 180° **(b)**.

Surfaces covered by a thin layer of nano-TiO_2_ do not become polluted even in the dark. Its particles are responsive also to water or pollutants and act as little ‘piles’. They push water drops and dust particles off the surface, hence preventing the surface from becoming wet or dirty. For the first time, this phenomenon was observed with *Nelumbo nucifera*, an angiosperm plant, and it was given the name lotus effect [85]. Surfaces displaying identical properties can also be found in animals, for example, the chitin armour of some insects, such as *Stenocara gracilipes* [86]. This phenomenon is referred to as superhydrophobicity [87] (Figure 3).

To summarize, surfaces covered by photocatalytic coatings that contain nanosized metal oxides, i.e. nano-TiO_2_, exhibit - when irradiated by UV - not only antistatic [33] or deodorizing [20] properties but, more importantly, they also have self-disinfecting [88,89] and self-cleaning abilities [13,66].

### Examples of TiO_2_/UV photocatalysis application in bacteria eradication

The majority of bacteria examined *in vitro* are common also in the natural environment, i.e. in water and soil, on the skin and in the digestive tract of humans and animals. Therefore, they may contaminate foods and may also form opportunistic biota in hospitals [90-96]. In recent years, numerous studies have been focused on the aspect of using the photocatalysis phenomenon in eradication of pathogenic microorganisms in ventilation systems [82,97-99], water supplies [27,29,32,82,100-103], sewage systems [104,105] and on work surfaces (e.g. tables and floors) in medical centres [15,16,106] or in food processing plants [107,108]. Among the photocatalytic processes that have been most studied in the context of microorganism inactivation and degradation of toxic compounds to human and animals is the TiO_2_/UV process where titanium dioxide (TiO_2_), after it has been powdered to nanoparticles, performs as photocatalyst, and ultraviolet (UV) radiation is an agent generating reactive oxygen species [10].

#### Air purification

Catalytic properties of nano-TiO_2_ exhibited in the presence of UV radiation give rise to its application, i.e. in decomposition of volatile organic compounds (VOC) [79], as well as in inactivation of microorganisms [99] present in building interiors of houses, offices or manufacturing sites [109]. Bioaerosols are nowadays the main air pollutant in rooms [98]. Many bacteria have been identified as infectious agents disseminated through ventilation systems. Among them, there are pathogens causing tuberculosis (*Mycobacterium tuberculosis*) [110-112], pneumonia (*Streptococcus pneumoniae*) [113], scarlet fever (*Streptococcus pyogenes*) [112,113], diphtheria (*Corynebacterium diphtheriae*) [112] or whooping cough (*Bordetella pertussis*) [114]. To purify air in closed areas, high efficiency particulate air (HEPA) filters are applied [115]. They arrest the majority of mechanical and biological pollutants. According to Goswami et al. [116], an equally effective tool in fighting the pathogens can be photocatalysis. For the purposes of the studies evaluating the effectiveness of the TiO_2_/UV process in inactivation of pathogens in closed areas, the authors designed UV-transparent air recirculation systems, covered by a thin nano-TiO_2_ layer. In this pioneering experiment, the air was completely free from microorganisms, and bacteria cells were entirely mineralized within a 5-h period of the TiO_2_/UV process [97]. Later experiments were able to reduce this period down to less than 3 min [98]. Photocatalytic processes, including the TiO_2_/UV process, are also an efficient tool in the eradication of bacteria spores, e.g. endospores of *Bacillus cereus* [117], as well as in eradication of microorganisms applied in biological weapons, e.g. *Bacillus anthracis* [118].

#### Water treatment

The TiO_2_/UV process finds its practical application also in water treatment [101]. The rapid growth of the human population in recent decades induces the increase in demand for drinking water. Incorrect management of water resources results in a pollution upsurge of world water resources, reflected by the growing number of waterborne disease outbreaks (WBDO) [119]. It might be attributed to the ineffective inactivation of pathogens in water treatment and water distribution systems and common disinfectants, such as chlorine, chlorine dioxide, chloramine or ozone [73]. Moreover, some pathogenic microorganisms, such as *Legionella pneumophila*, show limited susceptibility to traditional disinfectants like chlorine [120]. Conventional water treatment methods generate around 600 harmful disinfection by-products (DBPs), such as trihalomethanes or chlorophenols [78,121]. Those compounds are formed in chemical reactions between natural organic substances and compounds of disinfectants with strong oxidizing properties. Many DBPs show carcinogenic, mutagenic and teratogenic properties [122]. An ideal disinfectant should have inactivating properties towards many species of microorganisms, should not induce forming of DBPs, must not negatively impact human health and should be low cost, easy to handle and to keep, non-invasive for work tools and harmless to the environment. Some NMs, e.g. nano-TiO_2_, fulfil those requirements and can, therefore, be used in both water treatment [105,123] and disinfection of solid surfaces [71,124,125]. Already in the beginning of the 1980s in the twentieth century, Heller et al. [68] reported that a small amount of catalytically active TiO_2_ powder added to wastewater exposed to solar light purifies that water after some time, and the organic pollutants are decomposed into simple non-organic compounds. Due to mineralization of a number of organic and biological pollutions that takes place on surfaces covered by a thin layer of nano-TiO_2_, the application of the TiO_2_/UV process in the treatment of much polluted wastewater, i.e. in the treatment of water coming from resin plants, paper mills, dye-works and refineries [100,103], is being discussed, as well as its application in the decomposition of toxins released to water by cyanobacteria [126]. The TiO_2_/UV process can also be rendered useful in hospitals as a tool to control the dissemination of Legionnaires disease, a disease induced by *L. pneumophila*, e.g. in hot water distribution systems [127].

#### Disinfection of work areas and food packaging

Frequent and thorough disinfection of surfaces with the purpose to reduce the amount of bacteria and to prevent their dissemination is a necessity, e.g. in food processing plants, microbiological laboratories, veterinary medicine clinics and hospitals. Conventional disinfection methods, such as cleaning with chemical disinfectants, are work and time consuming and not always sufficient [128]. Photocatalytic processes taking place on surfaces covered by a thin layer on nanosized metal oxides (i.e., nano-TiO_2_) present an increasingly important alternative to traditional disinfection methods [97,102,104,129,130]. A reason for the greater interest in the practical application of photocatalysis is its high bactericidal effectiveness in the treatment of microorganisms, such as *Escherichia coli*, *Pseudomonas aeruginosa*, *Staphylococcus aureus* and *Enterococcus faecium* [71,129,131-134], playing a crucial role in public health protection.

According to Szczawiński et al. [107,108] as well as Tomaszewski and Jach [62], the number of living cells of *E. coli*, *P. aeruginosa*, *Salmonella* Enteritidis and *S. aureus* on the floor and wall ceramic tiles (applied in production halls, hospital rooms or sanitary rooms) covered by a thin layer of nano-TiO_2_ and exposed to UV radiation for 2 min was reduced by 6 to 7 logarithmic units, depending on the method of photocatalytical layer deposition and on the kind of tiles (shiny or matt). On control surfaces, the bacteria reduction rate amounted to around 1 logarithmic cycle.

The TiO_2_/UV process can also be applied in disinfection of food packaging. Its high efficiency in eradication of *E. coli* (strain ATCC11775) has been repeatedly proven [135], similar to the eradication of *Listeria monocytogenes* [136] from plastic bags and containers used for food storage.

The importance of effective surface disinfection in food manufacturing can hardly be overestimated. Shortcomings in this field have frequently caused various ingestions and poisonings. After an epidemic caused by *E. coli* (strain O157:H7) in Japan, the influence of the TiO_2_/UV process on the decomposition of the toxin produced by this bacterial strain was examined. The results showed that after 120 min of the TiO_2_/UV process, a partial decomposition of the toxin occurred, and after a further 2 h, its entire decomposition followed [33]. The studies by Oza et al. [137] confirmed high effectiveness of the TiO_2_/UV process not only in the decomposition of the toxin, but also in the inactivation of the bacteria producing the toxin. Guillard et al. [82] observed in their experiments on *E. coli* (strain PHL1273 with *curli*, a sort of long, flaccid adhesive fibres, a proteinaceous component of an extra-cellular matrix, formed on the surface of some bacteria species, allowing the bacteria to cling to the bottom) a reduction in the TBC by 7 logarithmic units after no more than 3 min of the TiO_2_/UV process.

As Szczawiński et al. [107,108] report, the application of photocatalytic surfaces, such as of ceramic wall tiles covered by a thin layer of nano-TiO_2_, in food processing plants, veterinary clinics, hospitals, treatment rooms, laboratories and wherever the UV radiation to disinfect surfaces is put to use, should significantly increase the disinfection effectiveness and contribute to a dramatic improvement of hygiene conditions in those areas. It has been pointed out for many years that photocatalytic processes can become a tool of a permanent surface disinfection of those items that people are in frequent contact with, e.g. door handles, taps or toilet seats [138]. Thereby, the disinfection process using AOPs can be broadly applied in the public areas, such as in toilets, schools, railway and bus stations, hotels, public transport and airports.

The TiO_2_/UV process does not only inactivate microorganisms, but also induces the mineralization of organic matter such as dead microorganisms [10,11]. Jacoby et al. [11] observed the inactivation of all cells of *E. coli* on a test ceramic surface after 30 min of the TiO_2_/UV process. After the next 45 min, 54% of the dead bacteria cells were completely oxidized to CO_2_ and H_2_O. In other experiments, the prolonged TiO_2_/UV process caused a complete mineralization of four strains of *L. pneumophila* (997, 1004, 1009, ATCC33153) [120]. Those studies have confirmed both the bactericidal properties and the self-cleaning properties of surfaces covered by a thin layer of nano-TiO_2_ and exposed to the UV radiation.

#### Disinfection of medical appliances

The AOP-based disinfection process has been tested also with regard to its feasibility in medicine [139]. Here, nano-TiO_2_ is used as a component of photocatalytic films covering catheters [140], scalpels [141] and surgical masks [142]. Ohko et al. [98] stated that the effectiveness of the UV-based disinfection of catheters is three times higher if they were covered by a thin layer of nano-TiO_2_. A similar result of the TiO_2_/UV application was observed with infected dental implants [143,144]. High bactericidal efficiency of the TiO_2_/UV process was also reported in orthopaedics and cosmetic surgery in the case of *S. aureus* on implants coated by a thin layer of nano-TiO_2_ [94]. It seems, therefore, photocatalytic processes can present a valuable method to reduce bacterial infections resulting from the application of implants in medicine.

## Conclusions

Nanotechnology allows modifying the properties of various materials through alteration of their structure at the level of atoms and molecules. Thus, products can be designed that are incomparably better than microtechnology products. Materials, components or devices designed using nanotechnology display a number of precious properties, i.e., they can be small, light, quick or efficient. Nanotechnology is regarded as the key technology of the twenty-first century. It appears as a source of new development opportunities for many economy sectors, and it may contribute to a better environmental protection. It can also assist in finding solutions to many problems in medicine, food hygiene and public health protection. TiO_2_ powdered to NPs is a material that, after it has been coated on surfaces and exposed to UV radiation, gives to those surfaces self-cleaning and self-disinfecting properties. This compound has, however, one important weakness: it absorbs UV radiation (*λ* < 400 nm), only that amounts to approximately 3% of the electromagnetic radiation spectrum that reaches the Earth. Thus, studies are already ongoing to examine the possibility of visible light (VL) (400 nm < *λ* < 700 nm) to excite nano-TiO_2_ that, if successful, would largely increase the effectiveness of photocatalysis and widen its application range. Photocatalytic processes, leading to inactivation of pathogens and to mineralization of organic pollutants on the surfaces coated by a thin layer of a photocatalyst, i.e. nano-TiO_2_, present a valid supplement of the traditional disinfecting methods. AOP-based disinfection is rapid and effective. Its application in the public space on a wide scale, especially where traditional disinfecting methods do not appear sufficient, can largely improve the sanitary and hygienic conditions, restrict the dissemination of pathogens, as well as reduce the number of food contaminations and poisonings. Photocatalytic processes help also keep the building walls clean for many years, road signs to not get dirty quickly and mirrors to not become covered by fog. It is supposed that nanomaterials - including nano-TiO_2_ - can play a crucial role, e.g. in medicine, food hygiene or public health protection. Will it be so, indeed? Let us wait and see. Nanotechnologists have not yet said their final ‘nano-word’.
